# A stochastic model for estimating sustainable limits to wildlife mortality in a changing world

**DOI:** 10.1111/cobi.13897

**Published:** 2022-04-28

**Authors:** Oliver Manlik, Robert C. Lacy, William B. Sherwin, Hugh Finn, Neil R. Loneragan, Simon J. Allen

**Affiliations:** ^1^ Biology Department, College of Science United Arab Emirates University Abu Dhabi United Arab Emirates; ^2^ Evolution and Ecology Research Centre, School of Biological Earth and Environmental Science University of New South Wales Sydney New South Wales Australia; ^3^ Species Conservation Toolkit Initiative Chicago Zoological Society Brookfield Illinois USA; ^4^ Curtin Law School, Faculty of Business and Law Curtin University Bentley Western Australia Australia; ^5^ Environmental and Conservation Sciences, College of Science, Health, Engineering and Education and Centre for Sustainable Aquatic Ecosystems, Harry Butler Institute Murdoch University Murdoch Western Australia Australia; ^6^ School of Biological Sciences University of Bristol Bristol UK; ^7^ Department of Anthropology University of Zurich Zurich Switzerland; ^8^ School of Biological Sciences University of Western Australia Perth Western Australia Australia

**Keywords:** conservation planning, dolphins, fisheries bycatch, PBR, population viability analysis, potential biological removal, PVA, SAMSE, análisis de viabilidad, AVP, captura incidental pesquera, delfines, EBP, extirpación biológica potencial, MASAM, planeación de la conservación, 保护规划, 海豚, 渔业副渔获物, 生物可移除潜在量(Potential Biological Removal, PBR), 种群生存力分析(PVA), 随机环境中的可持续人为影响死亡率(SAMSE)

## Abstract

Human‐caused mortality of wildlife is a pervasive threat to biodiversity. Assessing the population‐level impact of fisheries bycatch and other human‐caused mortality of wildlife has typically relied upon deterministic methods. However, population declines are often accelerated by stochastic factors that are not accounted for in such conventional methods. Building on the widely applied potential biological removal (PBR) equation, we devised a new population modeling approach for estimating sustainable limits to human‐caused mortality and applied it in a case study of bottlenose dolphins affected by capture in an Australian demersal otter trawl fishery. Our approach, termed *sustainable anthropogenic mortality in stochastic environments* (SAMSE), incorporates environmental and demographic stochasticity, including the dependency of offspring on their mothers. The SAMSE limit is the maximum number of individuals that can be removed without causing negative stochastic population growth. We calculated a PBR of 16.2 dolphins per year based on the best abundance estimate available. In contrast, the SAMSE model indicated that only 2.3–8.0 dolphins could be removed annually without causing a population decline in a stochastic environment. These results suggest that reported bycatch rates are unsustainable in the long term, unless reproductive rates are consistently higher than average. The difference between the deterministic PBR calculation and the SAMSE limits showed that deterministic approaches may underestimate the true impact of human‐caused mortality of wildlife. This highlights the importance of integrating stochasticity when evaluating the impact of bycatch or other human‐caused mortality on wildlife, such as hunting, lethal control measures, and wind turbine collisions. Although population viability analysis (PVA) has been used to evaluate the impact of human‐caused mortality, SAMSE represents a novel PVA framework that incorporates stochasticity for estimating acceptable levels of human‐caused mortality. It offers a broadly applicable, stochastic addition to the demographic toolbox to evaluate the impact of human‐caused mortality on wildlife.

## Abstract 2

*Article impact statement*: The new SAMSE model incorporates stochasticity to provide better estimates of sustainable limits to wildlife mortality in a changing world.

## Resumen

La mortalidad de la fauna causada por humanos es una amenaza continua para la biodiversidad. El análisis del impacto a nivel poblacional de la captura pesquera incidental y otras causas humanas de la mortalidad de la fauna comúnmente ha dependido de métodos determinísticos. Sin embargo, las declinaciones poblacionales con frecuencia se aceleran por los factores estocásticos que no son considerados en dichos métodos convencionales. A partir de la ecuación de extirpación biológica potencial (EBP) de extensa aplicación diseñamos una nueva estrategia de modelación poblacional para estimar los límites sustentables de la mortalidad causada por humanos y la aplicamos en un estudio de caso de los delfines nariz de botella afectados por la captura en una pesquería australiana de arrastre demersal. Nuestra estrategia, denominada *mortalidad antropogénica sustentable en ambientes estocásticos* (MASAM) incorpora la estocasticidad ambiental y demográfica, incluyendo la dependencia que tienen las crías por sus madres. El límite MASAM es el número máximo de individuos que pueden extirparse sin causar un crecimiento poblacional estocástico negativo. Calculamos un EBP de 16.3 delfines por año con base en la mejor estimación de abundancia disponible. Como contraste, el modelo MASAM indicó que sólo podían extirparse entre 2.3 y 8.0 delfines anualmente sin ocasionar una declinación poblacional en un ambiente estocástico. Estos resultados sugieren que las tasas reportadas de captura incidental no son sustentables a largo plazo, a menos que las tasas reproductivas sean sistemáticamente más altas que el promedio. La diferencia entre el cálculo determinístico del EBP y los límites de MASAM mostró que los enfoques determinísticos pueden subestimar el verdadero impacto de la mortalidad de la fauna causada por humanos. Lo anterior resalta la importancia de integrar la estocasticidad al evaluar el impacto de la captura incidental y otras causas humanas de la mortalidad como la caza, las medidas letales de control y las colisiones con turbinas de viento. Aunque el análisis de viabilidad poblacional (AVP) se ha utilizado para evaluar el impacto de la mortalidad causada por humanos, MASAM representa un marco novedoso de AVP que incorpora la estocasticidad para estimar los niveles aceptables de mortalidad causada por humanos. Este enfoque ofrece una adición estocástica de aplicación generalizada para las herramientas demográficas usadas para evaluar el impacto de la mortalidad causada por humanos sobre la fauna.

## 【摘要】

在不断变化的情景下估计野生动物死亡率的持续性限度的随机模型

人类造成的野生动物死亡是对生物多样性的普遍威胁。目前, 通常依赖于确定性方法来评估渔业副渔获物和其它人为造成的野生动物死亡在种群水平上的影响。然而, 随机因素往往会加速种群数量的下降, 但这种常规方法没有将其纳入考虑。我们在已得到广泛应用的生物可移除潜在量(Potential Biological Removal, PBR)方程的基础上, 设计了一种新的种群建模方法来估计人为造成的死亡的可持续限度, 并将其应用于澳大利亚底层拖网渔业中受影响的瓶鼻海豚的案例研究。我们的方法可以称为“随机环境中的可持续人为影响死亡率(SAMSE)”, 包含了环境和种群的随机性, 还包括了后代对母亲的依赖性。SAMSE的极限是指在不引起负面的种群随机增长的情况下可以移除的最大个体数。我们根据现有最佳的丰度估计, 计算出海豚的生物可移除潜在量为每年16.2头。相比之下, SAMSE模型则显示, 在随机环境中, 为了不引起种群下降, 每年只能移除2.3至8.0头海豚。这些结果表明, 除非繁殖率持续高于平均水平, 否则长期来看报告的副渔获率是不可持续的。确定性的PBR计算和SAMSE限制之间的差异表明, 确定性方法可能低估了人为造成野生动物死亡的真实影响。这突出了整合随机性在评估副渔获或其它人为造成的死亡(如狩猎、灭杀控制措施和风力涡轮机碰撞)对野生动物的影响中的重要性。尽管种群生存力分析(PVA)已被用于评估人为造成死亡的影响, 但SAMSE代表了一个包含随机性的PVA新框架, 可以用于估计人为造成死亡的可接受水平。它为种群统计学工具补充了一种广泛适用的随机性方法, 以评估人类造成的死亡对野生动物的影响。**【翻译:胡怡思;审校:聂永刚**】

## INTRODUCTION

Mortality caused by incidental capture, or bycatch, in commercial fisheries is an ongoing, global problem for conservation and sustainable fisheries management (Read et al., 2006). A commonly used tool to evaluate the effect of fisheries bycatch is the potential biological removal (PBR) equation (Wade 1998). The PBR is used to estimate the maximum number of animals that may be removed from a “stock” while allowing that stock to reach or maintain its “optimum sustainable population” based on an abundance estimate, a population growth rate, and a recovery factor (Wade 1998). The PBR is considered to provide a conservative limit for human‐caused mortality (Moore et al., 2009). The U.S. Marine Mammal Protection Act (MMPA) of 1972 provides the statutory framework for the PBR concept (Moore et al., 2013). The MMPA was amended in 1994 to include a requirement that the level of incidental mortality and serious injury to marine mammals be reduced to insignificant levels approaching a zero rate, commonly referred to as the zero mortality rate goal (ZMRG). Regulators in the United States determined that the threshold level for the ZMRG would be 10% of the PBR for a stock (NOAA 2004). PBR incorporates uncertainty by using minimum population size (*N*) estimates and including an adjustable recovery factor (Wade 1998). However, this original analysis framework used to compute the default parameters of PBR is based on a deterministic control rule. In other words, the original PBR calculation does not account for demographic or environmental stochasticity, differences in life stages, or the influence of fluctuating reproductive rates.

Although deterministic processes typically initiate population declines, the final steps toward extinction are often driven by a myriad of stochastic factors (Gilpin & Soulé 1986; Lande 2002). Given that many conventional approaches, including the original PBR equation, do not account for stochasticity, evaluation of the true impact of human‐caused mortality requires a stochastic tool. Wade (1998, p. 27) recognized this and, upon introducing PBR, suggested that it required supplementation by evaluating “the effects of stochastic dynamics.” Likewise, Roughgarden and Smith (1996) called for fisheries to attain “ecological stability,” which, in a variable and uncertain environment, would require harvesting at less than the maximum sustainable yield. Over time, the original PBR operating model was relaxed to also account for different life stages and demographic or some aspects of environmental stochasticity. For example, Brandon et al. (2017) and Punt et al. (2018) incorporated stochasticity into PBR models by varying estimates for vital rates and varying *N* based on observation error. However, these studies did not include environmental variance (EV)—variation in demographic rates over time—which has a large effect on the viability of slow‐growing animal populations (Manlik 2019). Punt et al. (2020a) used a stochastic population model, including EV, to test the robustness of the PBR, demonstrating that sources of stochasticity can cause the PBR to result in misleading estimates of sustainable removals. They, therefore, suggest that the parameters in the PBR equation should be adjusted downward for species with considerable variation in demographic rates. However, many still rely on the original PBR equation to set bycatch limits without incorporating stochasticity, which is less suitable for threatened species that usually have small to moderate population sizes (Wade 1998). We built on previous research and propose a new methodological framework that incorporates stochasticity for estimating sustainable levels of human‐caused mortality.

In combination with sensitivity analyses, population viability analysis (PVA) is a useful management tool because it can be used to identify parameters critical to population dynamics and to evaluate the impacts of specific threats (Manlik et al., 2018). PVA allows the integration of demographic and environmental stochasticity (Lacy 2019), and has been used to assess the effect of fisheries bycatch on nontarget species (e.g., Cervin et al., 2020). However, PVA is not typically used to set acceptable levels of bycatch or other human‐caused mortality. We found only 1 study that used a similar PVA approach to estimate acceptable harvest levels for dugongs (*Dugong dugon*) in Australia (Heinsohn et al., 2004). The authors concluded that “no more than 100 dugongs per annum” should be taken because all scenarios based on higher harvest levels forecast population declines. However, that approach did not provide a specific harvest threshold, akin to PBR, because only 4 levels of human‐caused mortalities were tested. Heinsohn et al. (2004) also did not devise a standardized approach. Hence, a standardized PVA framework that incorporates environmental and demographic stochasticity for estimating sustainable levels of harvest is still lacking. Using a comprehensive stochastic modeling approach in setting human‐caused mortality limits would thus be an important new application of PVA.

We assessed the impact of bycatch in the Pilbara Fish Trawl Interim Managed Fishery (PTF) off northern Western Australia. The PTF is a demersal, otter trawl fishery that targets a variety of species, including emperors, snappers, trevally, and cods (Department of Fisheries, Western Australia 2010). It also captures nontarget protected species, including bottlenose dolphins (*Tursiops truncatus*) (Jaiteh et al., 2014). Independent fishery observers estimated bycatch mortality rates of 45–60 dolphins per year from 2003 to 2009 (Allen et al., 2014). Observer‐reported dolphin bycatch rates were thus 2–3 times those reported by skippers (17–34 dolphins per year, 2006–2009), and likely an underestimate because some dolphins were caught and then expelled via bycatch reduction devices before being landed on deck (Allen et al., 2014; Jaiteh et al., 2013, 2014; Wakefield et al., 2014). Observer coverage was discontinued after 2009, but skipper‐reported bycatch rates of 16–33 dolphins per year from 2010 to 2017 were indicative of the ongoing trend (Fletcher & Santoro 2012; Gaughan et al., 2019). This dolphin population is genetically isolated from populations inshore of the PTF (Allen et al., 2016) and includes dolphins that show fidelity to fishery‐associated foraging (Allen et al., 2017).

We devised a stochastic modeling approach to evaluate the effect of bycatch on the dolphin population interacting with the PTF. The approach is also intended to provide a general tool for estimating limits for human‐caused mortality in stochastic environments. We aimed to estimate a bycatch limit corresponding to population stability, whereas approaches such as PBR aim for population recovery. Additionally, our approach incorporated stochasticity, variation in reproductive rates, and dependency of offspring on the fate of their mothers. We performed a PVA in vortex (Lacy et al., 2014), which has previously been used to forecast the population viability of various taxa, including dolphins (e.g., Lacy et al., 2017; Thompson et al., 2000; Vermeulen & Bräger 2015).

The overall objective was to provide a novel approach that incorporates demographic and environmental stochasticity in the evaluation of the impact of human‐caused mortality on affected populations, offering a tool to estimate a limit to human‐caused mortality (i.e., SAMSE). We had 4 specific aims. The first aim was to assess the effect of skipper‐reported and independently observed dolphin bycatch mortality levels in the PTF with a stochastic PVA model. Second, we aimed to calculate PBR and assess the effect of human‐caused mortality at the PBR level with a stochastic PVA modeling approach. Third, we aimed to determine the sustainable mortality limit in stochastic environments (SAMSE limit), which we defined as the maximum number of individuals that can be removed from the population without causing a negative stochastic population growth. The fourth aim was to compare predictions of PBR, SAMSE, and incidental dolphin capture rates. Moreover, we aimed to construct SAMSE so that it could be readily tailored to a range of taxa and human–wildlife conflicts in stochastic environments. We defined human‐caused mortality of wildlife as any human actions that cause mortality of wildlife that could affect a population's viability, regardless of whether this mortality is deliberate, incidental, unlawful, or authorized.

## METHODS

Our PVA model integrated dolphin abundance estimates for the affected population with vital rates from a congeneric reference population (*Tursiops aduncus*) in Shark Bay, Australia, which is projected to have positive (although small) population growth in the absence of bycatch (Manlik et al., 2016). *Tursiops* congeners share common vital rates (Taylor et al., 2007). The surrogate population has been closely researched over 4 decades (e.g., Connor & Smolker 1985; King et al., 2021; Manlik et al., 2019;) and was estimated to be similar in size to the PTF‐associated population (Manlik et al., 2016).

### PBR and its effect on population viability

We calculated the number of animals that could be removed from the population (not including natural mortality) per year while reaching or maintaining an optimum sustainable population. We used the formula PBR = *N*_min_ (*R*_max_/2) *F*_R_, where *N*_min_ is a minimum population size estimate; *R*_max_ is conventionally assumed to be 0.04 for cetaceans, being the population growth rate at low densities; *R*_max_/2 corresponds deterministically to the lower limit of an optimal sustainable population (OSP) for the operating model applied by Wade (1998); and *F*_R_, the recovery factor, is set to a default 0.5 (Wade 1998). The *N*_min_ is based on the abundance estimate for the PTF‐associated dolphin population (Allen et al., 2017) and takes the lower 95% confidence interval (CI) limit (*N* = 1247) corrected upward by an availability factor (0.77) estimated for the same species in a similar environment (Forcada et al., 2004) for an *N*_min_ of 1619 (1247 divided by 0.77).

### Population viability analysis

To assess the effect of fisheries bycatch on the viability of the PTF‐associated dolphin population in the presence of stochastic events, we conducted a PVA with vortex version 10 (www.scti.tools/vortex) (Lacy et al., 2014). Unlike conventional matrix models, vortex is an age‐based Monte Carlo simulation that integrates the interacting effects of deterministic and stochastic processes that influence the viability of populations (Lacy 1993, 2000). Lacy (2000) details information on the structure of the vortex program. Given its small positive population growth (Manlik et al., 2016), the Shark Bay dolphin population may be close to carrying capacity. Consequently, it may experience a density‐dependent effect, such that growth would be faster if the population declines. However, no evidence for such density dependence was detected in another bottlenose dolphin population that is believed to be approaching carrying capacity (Lacy et al., 2021). Lacking data with which to estimate the magnitude or even shape of density dependence, we used the default ceiling model in vortex.

### Incorporating demographic and environmental stochasticity

**FIGURE 1:**
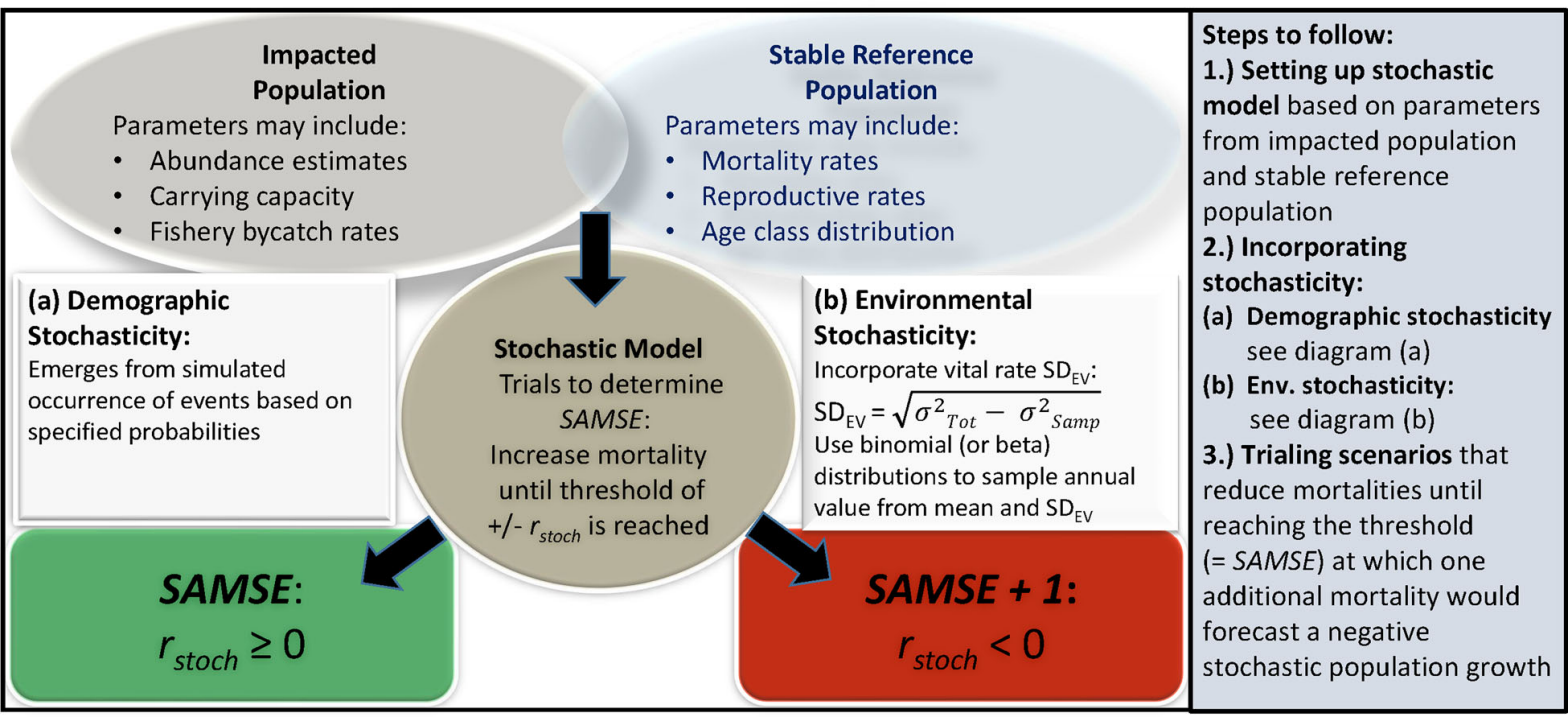
A general population viability analysis framework to determine the *sustainable anthropogenic mortality in stochastic environments* (SAMSE) with stochastic modeling on the basis of input parameters from affected populations and a reference population. The SAMSE limit is the maximum number of individuals that can be removed without causing negative stochastic population growth (*r*_stoch_); SAMSE + 1 = the maximum number of individuals that can be removed, plus 1 additional removal, resulting in negative stochastic growth rates (*r*_stoch_). For the formula to calculate SDs due to environmental variance for mortality and reproductive rates, measured in several time periods, *σ* ^2^_tot_ is the total temporal variance across the data, and *σ* ^2^_samp_ is the mean sampling (binomial) variance of rates for individual time periods

We incorporated demographic and environmental stochasticity in our PVA model. We used 3‐year rates because the life history for this species is such that 3‐year age classes match well with the demography (Manlik et al., 2016). Combining across yearly age classes, such as this, the random sampling noise in estimating demographic rates is reduced, thereby typically providing better estimates of the demographic rates for the modeling. However, it is not necessary to use 3‐year rates—any apt time interval can be used in vortex. Environmental stochasticity is the temporal variation in probabilities of vital rates due to random fluctuations in the environment. vortex incorporates environmental variation in reproductive and mortality rates by sampling population rates each year from user‐specified distributions. For this purpose, we applied the 3‐year standard deviations due to EV (SD_EV_) for the age‐specific mortality and reproductive rates, as reported for Shark Bay (Manlik et al., 2016). Under an assumption of random sampling and independent fates of individuals each year, the total observed variance in each vital rate is the sum of the sampling (binomial) variance and the variance due to environmental fluctuations (Figure 1). Therefore, the SD_EV_s were calculated by Manlik et al. (2016) by subtracting the expected sampling variance from the total observed temporal variance, following Akçakaya (2002) and Miller and Lacy (2005).

In contrast, demographic stochasticity is the random fluctuation in observed vital rates and sex ratios due to stochastic sampling processes, even if the probabilities of these rates remain constant over time (Lacy et al., 2014). Demographic stochasticity is incorporated by determining the fate (survival and reproductive outcomes) of each individual as Bernoulli processes using the mean rates for the population in a given period (Lacy 2000). Specifically, vortex incorporates demographic stochasticity by setting the occurrence of probabilistic events, such as reproduction and sex ratios, with a pseudorandom number generator. For each life event, if the random value sampled from a specified distribution falls above the specified probability, the event is considered to have occurred, which triggers a Bernoulli process. Hence, demographic stochasticity is the result of the uncertainty whether a specific demographic event occurs for any given individual. Additionally, we incorporated stochasticity by modeling the calves’ dependency on the fate of their mothers (see “Bycatch scenarios”).

### Baseline scenarios

As a no‐bycatch baseline scenario for comparison with scenarios of varying levels of bycatch, we used a PVA model of the Shark Bay bottlenose dolphin population (Manlik et al., 2016). This population is stable, with comparatively high reproductive rates and consistently high survival rates over 12 years in the absence of bycatch (Manlik et al., 2016). We set up no‐bycatch scenarios using all demographic parameters (Manlik et al., 2016; Table 1; 3‐year mortality rates and initial age classes in Appendices S1 and S2), except for *N* and carrying capacity (*K*). We set up 3 baseline scenarios with differing initial population size estimates (*N*_0_) (Allen et al., 2017), with the availability correction factor of 0.77 as for *PBR*: *N*_0_ = 2953 (best estimate of 2274 divided by 0.77), 1619 (lower 95% CI 1247 divided by 0.77), and 5473 (upper 95% CI 4214 divided by 0.77). Without reliable information, such as resource availability or competition, to estimate *K* accurately, *K* for all scenarios was set to 10,946 or double the adjusted upper 95% CI for abundance to allow for population expansion.

**TABLE 1:** Population forecasts for an initial population size (*N*_0_) of 2953 (best estimate), 5473, and 1619^a^

		No bycatch	Bycatch 1^b^	Bycatch 2^c^	Bycatch 3^d^	Maximum bycatch^e^	Potential biological removal (+48.57 per 3‐year period)
***N*_0_ = 2953**							
Stochastic growth rate (SE)		0.0055^f^ (0.0001)	–0.0115 (0.0001)	–0.0262 (0.0001)	–0.0650 (0.0003)	–0.1002 (0.0004)	–0.0154 (0.0004)
Time to extinction (years)		NA	NA	NA	270.3	206.4	NA
63.3‐year forecast^h^	*N*_63.3_ (SE)	3496.6 (18.8)	2483.9 (14.3)	1838.0 (10.2)	909.0 (5.5)	446.5 (2.8)	2254.9 (13.0)
	% change	18.4	–15.9	–37.8	–69.2	–84.9	–23.6
100‐year forecast	*N*_100_ (SE)	3694.6 (25.2)	2165.6 (16.7)	1344.3 (9.9)	440.8 (3.7)	142.2 (1.4)	1874.0 (14.2)
	% change	25.1	–26.7	–54.5	–85.1	–95.2	–36.5
***N*_0_ = 5473**							
Stochastic growth rate (SE)		0.0056 (0.0001)	–0.0034 (0.0001)	–0.0116 (0.0001)	–0.0294 (0.0001)	–0.0486 (0.0001)	
Time to extinction (years)		NA	NA	NA	NA	290.4	
63.3‐year forecast^h^	*N*_63.3_ (SE)	6421.0 (31.2)	4698.0 (26.6)	4575.4 (24.3)	3168.4 (17.3)	2174.1 (12.5)	
	% change	17.3	–14.2	–16.4	–42.1	–60.3	
100‐year forecast	*N*_100_ (SE)	6688.5 (37.4)	4303.2 (27.5)	3978.9 (28.0)	2220.8 (16.4)	1224.3 (9.7)
	% change	22.2	–21.4	–27.3	–59.4	–77.6
***N*_0_ = 1619**							
Stochastic growth rate (SE)		0.0054 (0.0001)	–0.0241 (0.0001)	–0.0603 (0.0003)	–0.1211 (0.0005)	–0.1834 (0.0007)	
Time to extinction (years)		NA	NA	267.2	156.9	104.1	
63.3‐year forecast^h^	*N*_63.3_ (SE)	1918.8 (10.2)	1076.0 (6.2)	580.2 (3.6)	154.1 (1.1)	38.8 (0.4)	
	% change	18.5	–33.5	–64.2	–90.5	–97.6	
100‐year forecast	*N*_100_ (SE)	2054.0 (14.1)	811.8 (6.2)	303.8 (2.6)	37.6 (0.5)	5.9 (0.1)	
	% change	26.9	–49.9	–81.2	–97.7	–99.6	
	PE^g^ (%)	0.0	0.0	0.0	0.0	47.1	

^a^ Three‐year stochastic growth rates (*r*_stoch_), percentage change in *N*, and mean time to extinction are shown for all scenarios based on mean reproductive rates.

^b^ Capture rate of 13 dolphins/year (39 per 3‐year period) for 2008 based on Department of Fisheries, Western Australia (2010).

^c^ Mean capture rate of 24.5 dolphins/year (73.5 per 3‐year period) for 2012–2017 based on skippers’ logbooks (Gaughan et al., 2019).

^d^ Mean capture rate of 50 dolphins/year (150 per 3‐year period) for 2002 and 2006–2009 based on independent observer reports (Stephenson & Chidlow 2003; Allen et al., 2014).

^e^ Hypothetical maximum catch rate of 75 dolphins/year (225 per 3 year‐period) based on statement by the Western Australian Department of Fisheries that the “number of dolphins caught by the fishery should be <75/year” (Fletcher & Santoro 2010, p. 313).

^f^ Three‐year stochastic growth rate (*r*_stoch_) based on calf‐dependent model. The 3‐year *r*_stoch_ based on model that does not assume that calves are dependent on their mothers was 0.0056.

^g^ Probability of extinction. It shows the percentage of iterations for which the population was forecast to go extinct. The PE was not tabulated for *N*_0_ = 2953 and *N*_0_ = 5473 and the 63.3‐year forecast at *N*_0_ = 1619, for which PE = 0% for all forecasts.

^h^ Forecasts for 3 *Tursiops* spp. generations (Taylor et al., 2007).

Baseline and bycatch scenarios simulated the population trajectory with a 3‐year interval, which corresponds to biologically meaningful age classes for the taxon and to the interval over which vital rates were estimated (Manlik et al., 2016). Each scenario was repeated for 1000 iterations (replications). We recorded forecasts for mean extant population sizes (*N*_63.3_; *N*_100_), probabilities of extinction (PE_63.3_, PE_100_), and 3‐year stochastic growth rates (*r*_stoch_) after 63.3 years (3 bottlenose dolphin generations [Taylor et al., 2007]) and 100 years. To report forecasts for noninteger number of years (e.g., 63.3‐year forecasts), we calculated a weighted mean that corresponds to the noninteger number based on the output values for integer number of years above and below that mean. The probability of extinction was defined as the percentage of simulations that forecast a population extinction (i.e., the absence of either sex). For this model, we assumed there was no migration, which is supported by genetic data that show the PTF‐associated population exhibits limited gene flow with its adjacent populations (Allen et al., 2016). Three‐year mortality rates and other parameters for the model are in Appendices S1 and S2, respectively.

### Bycatch scenarios

We set up 3 bycatch scenarios (bycatch 1, 2, and 3) by modeling an increase in age‐specific mortality rates relative to the baseline. For bycatch 1, we used the skippers’ logbook‐reported capture of 13 dolphins in 2008 (Department of Fisheries, Western Australia 2010) (i.e., a 3‐year capture rate of 39 dolphins, the lowest reported rate in the last decade for the PTF). Bycatch 2 was based on skippers’ logbook reports in the PTF from 2012 to 2017 (Fletcher & Santoro 2013; Gaughan et al., 2019), at a mean capture rate of 24.5 individuals per year or a 3‐year capture rate of 73.5 individuals. For bycatch 3, we used dolphin capture rates of 50 per year, as reported by independent observers in 2002 (Stephenson & Chidlow 2003) and similar to observer‐reported rates from 2006 to 2009 (Allen et al., 2014), or a 3‐year capture rate of 150 dolphins. We also set up 1 max bycatch scenario based on a hypothetical maximum catch rate derived from the statement by the then Western Australian Department of Fisheries (DoF) that the “number of dolphins caught by the fishery should be <75/year, assuming 100% catch mortality” (Fletcher & Santoro 2010, p. 313) (3‐year capture rate 225 dolphins). For each bycatch scenario, we assumed that the chance of bycatch was the same for all age classes. Thus, we adjusted calf, juvenile, and adult mortalities by adding the respective number of bycatch mortalities to the number of mortalities of the baseline scenarios (Appendix S3). We calculated age‐class‐specific mortality rates by dividing the adjusted mortalities by the total number of individuals per age class and 3‐year interval according to the age class distribution of the Shark Bay population (Manlik et al., 2016). For the bycatch scenarios, we kept the SD_EV_s for corresponding mortality rates without bycatch because the observed variance of mortality rates is only related to natural mortality. An additional vortex scenario tested the effect of bycatch at the calculated PBR level of 16.19 dolphins/year, adding 48.57 mortalities per 3 years to the baseline 3‐year mortality. As per the other scenarios, we assumed that bycatch randomly affects all ages. The PBR scenario used the best estimates of reproductive rates (58.35% of adult females breeding per 3‐year period) and population size (*N*_0_ = 2953 [Allen et al., 2017]).

Bottlenose dolphin calves are dependent on their mothers until weaned (Mann et al., 2000; Wells et al., 1987). A mother's death in a net thus means its calf is unlikely to survive, either because it is also caught due to its close association with its mother or because it will be unable to survive on its own. Furthermore, the close association between mother and calf negatively affects maternal swim performance, plausibly rendering dolphin mothers more vulnerable to bycatch (Noren 2013). We therefore adjusted the vortex model to reflect the calves’ dependency on their mothers. Under this setting, the model removes any calves whose mothers die but ignores whether sires are alive or not. The settings for these calf‐dependent scenarios are explained in Appendix S3; adjusted calf mortalities for dependent‐calves are in Appendix S1.

### High and low reproductive rate scenarios

To evaluate the effect of fluctuations in reproductive rates on population dynamics, we set up additional scenarios based on observed temporal variation in reproductive rates reported for the Shark Bay population. Over 12 years, the mean percentage of adult females breeding in 4 3‐year periods was 58.4% (SD_EV_ = 9.38) (Manlik et al., 2016). We used the standard errors of the mean (±5.6) to set up scenarios of high and low reproductive rates (i.e., 64.0% and 52.8% of adult females breeding in each 3‐year period). There were 36 scenarios, or 4 dolphin capture rates (including the no‐bycatch scenarios) × 3 *N*_0_ estimates × 3 reproductive rates.

### Assessing effect of stochasticity on forecasts

To assess the effect of stochasticity on model forecasts, we ran additional simulations and compared the 3‐year population growth rate forecasts for all bycatch scenarios (bycatch 1–3, max bycatch) and population size estimates (*N*_0_ = 2953; *N*_0_ = 5473; *N*_0_ = 1619), based on the following. First, we set up a fully stochastic model with environmental and demographic stochasticity and calf dependency (as described above). Second, we simulated lowered environmental stochasticity, relative to the fully stochastic model, by setting the SD_EV_ for the percentage of adult females breeding to 0 (SD_EV_ = 0 for reproduction). Third, the simulated influence of environmental stochasticity on mortality was reduced by setting the SD_EV_ for each age‐specific mortality rate to 0 (SD_EV_ = 0 for mortality). Fourth, we simulated lower levels of environmental stochasticity relative to the others by setting SD_EV_s for the percentage of adult females breeding and all age‐specific mortality rates to 0 (SD_EV_ = 0 for reproduction and mortality). Finally, we performed a deterministic calculation in vortex, which is based on a standard life‐table analysis that determines the mean population growth rate in the absence of all stochastic variation (Lacy et al., 2014). The deterministic calculations were based on the settings that assume that calves are not dependent on the mother because the calculation does not account for the state variable, but also because we wanted to compare the fully stochastic forecasts with entirely deterministic forecasts. All of these comparisons were based on the mean reproductive rates. Additionally, we compared 3‐year stochastic growth rates based on the dependent‐calf settings versus the independent‐calf settings for all bycatch scenarios with *N*_0_ = 2953.

### Sustainable anthropogenic mortality in stochastic environments

We defined the SAMSE limit as the maximum number of individuals that can be removed by human activity, in this case fisheries bycatch, per year (or every 3 years) without resulting in negative stochastic growth rate forecasts (Figure 1). By this definition, removing 1 more individual per year would result in a population decline (i.e., a negative stochastic growth rate, *r*_stoch_). To estimate the SAMSE limit, we conducted trials based on the scenarios that incorporated stochastic variation. We ran scenarios that included the removal of a set number of individuals until we reached forecasts that produced nonnegative stochastic growth rates. Hence, the number of individuals removed in those scenarios that produced nonnegative growth rates was defined as the SAMSE limit. We also recorded SAMSE + 1 to show growth rate projections for the maximum number of individuals that could be removed, plus 1 additional removal, resulting in negative stochastic growth rates. We determined SAMSE limits for 6 scenarios based on all 3 population size estimates (*N*_0_ = 1619, *N*_0_ = 2953, *N*_0_ = 5473) × 2 reproductive rates (mean, ± 1 SE). We also recorded 3‐year deterministic growth rates (*r*_det_) for scenarios based on the assumption that calf mortalities are independent of their mothers’ fate in order to compare the effect on stochastic versus deterministic growth rate projections.

## RESULTS

**FIGURE 2:**
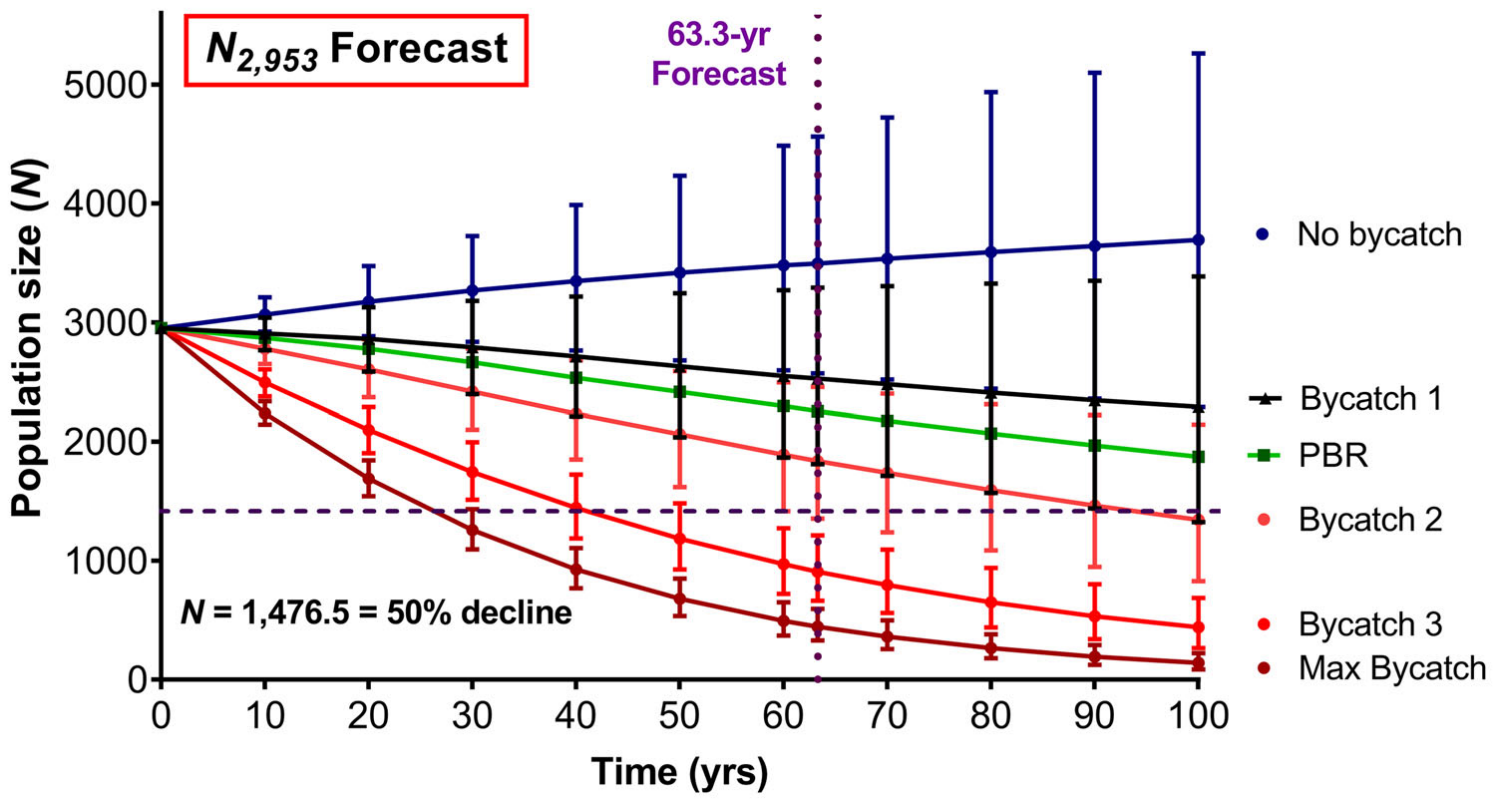
Dolphin population trajectories based on 6 scenarios: no bycatch‐related mortalities; bycatch scenarios based on 39, 73.5, 150, 225, and 48.57 (PBR = potential biological removal) dolphin captures per 3‐year period generated from models with initial population size (*N*_0_) of 2953. Thirteen dolphins/year (39 per 3‐year period) is the mortality rate for 2008 based on Department of Fisheries, Western Australia (2010) (bycatch 1); 24.5 dolphin/year (73.5 per 3‐year period) is the mean capture rate (2012–2017) based on skippers’ logbooks (Fletcher & Santoro 2013; Gaughan et al., 2019) (bycatch 2); 50 dolphins/year (150 per 3‐year period) is the mean capture rate (2002 and 2006–2009) based on independent observer reports (Stephenson & Chidlow 2003; Allen et al., 2014) (bycatch 3); 75 dolphin/year (225 per 3 year‐period) is the hypothetical maximum catch rate based on a statement by the Western Australian Department of Fisheries that the “number of dolphins caught by the fishery should be <75/year” (Fletcher & Santoro 2010, p. 313) (max bycatch). Whiskers show results for high (SE +1) and low (SE –1) reproductive rates (Manlik et al., 2016)

The vortex baseline scenario, based on mean Shark Bay bottlenose dolphin vital rates without mortalities from bycatch, forecast a stable population not at risk of extinction (Figure 2; Table 1). The 3‐year stochastic population growth rate (*r*_stoch_) for the baseline model, based on the assumption that calf mortality is dependent upon the fate of their mothers, was 0.0055. The 3‐year *r*_stoch_, based on the assumption that the fate of calves is independent of that of their mothers, was 0.0056. This slightly positive rate resembles that of the Shark Bay population (Manlik et al., 2016). Population declines for no‐bycatch scenarios were forecast only in scenarios with lower‐than‐average reproductive rates (Figure 2; Appendices S4 & S6).

### Stochastic forecasts based on bycatch scenarios

In contrast to the baseline scenario, all bycatch scenarios in vortex based on mean reproductive rates projected negative population growth (Figure 2; Table 1). Based on best estimates for *N* and reproduction, the forecast 3‐year population growth rates for bycatch scenarios 1, 2, 3, and the maximum bycatch rate, with annual capture rates of 13, 24.5, 50, and 75 individuals, respectively, were –0.0115, –0.0262, –0.0650, and –0.1002 (Table 1). The population was forecast to decline by 55% after 100 years if 24.5 individuals were removed per year (bycatch 2; Table 1). Bycatch 3 and max bycatch scenarios resulted in >50% declines after 3 generations (63.3 years; Table 1). Other than bycatch 1, all bycatch scenarios with low abundance (1619) forecast declines of >50% after 3 generations (Table 1). With high abundance (5473), only annual removal of 75 individuals (max bycatch) would result in a decline of >50% after 3 generations (Table 1).

The extinction probability was 0% for all scenarios based on the best abundance estimate (2953) and mean reproduction, but it was 47% for the max bycatch scenarios after 100 years with the low abundance estimate (Table 1). All scenarios based on lower‐than‐average reproduction forecast population declines (Appendix S5). Carrying capacity was not reached for any scenario.

### PBR and its effect on stochastic model forecast

The deterministic PBR estimate for the dolphin population was 16.19 individuals. Thus, 16.19 dolphins could be removed from the population per year (or 48.57 per 3‐year period), so that the population would be able to rebuild in the absence of stochastic events. Subsequently, we investigated whether an annual PBR of 16.19 was realistic by applying this value to the baseline vortex model (best abundance estimate, mean reproduction). The removal of 48.57 individuals per 3‐year period forecast a population decline by over one‐third after 100 years, and the 3‐year *r*_stoch_ was –0.0154 (Table 1). The forecast decline after 3 generations was 24%, and the probability of extinction for the *PBR*‐simulated forecast was 0% for any given time period (Table 1).

### Effect of stochasticity on model forecasts

The comparison between the fully stochastic model and the deterministic calculations showed that adding stochasticity to the model substantially reduced the forecast population growth rates (Appendices S7 & S8). Forecast growth rates for the bycatch scenarios of the fully stochastic model were, on average, 16.9–22.6% lower than those derived from deterministic calculations, depending on *N*_0_ (Appendix S7). For the best population size estimate, *N*_2953_, the mean 3‐year *r* across all bycatch scenarios was reduced from – 0.0415 (*r*_det_) to – 0.0507 (*r*_stoch_) when adding environmental and demographic stochasticity, including the calf's dependency on the mother's fate (Appendix S7). Setting SD_EV_ for reproductive rates to 0 resulted in 3‐year growth rates that were 2.6–6.8% lower than the average deterministic growth rates, depending on *N*_0_ (Appendix S7). Likewise, setting SD_EV_ for mortality rates to 0 resulted in 2.1–6.8% lower population growth compared with *r* derived from deterministic calculations (Appendix S7). The 3‐year population growth rates for bycatch scenarios that incorporated the calf's dependency on the mother's fate were, on average, 13.4% lower than the 3‐year *r* that was forecast for the less stochastic, independent‐calf model (dependent calf: *r* = –0.0507; independent calf: *r* = – 0.0447) (Appendix S8).

### Sustainable anthropogenic mortality in stochastic environments

Based on the best abundance estimate, the maximum number of individuals that could be removed from the population without resulting in a negative stochastic growth rate was 4.33 per year (SE = 0.0001), or 13 per 3‐year period (Table 2). One additional removal per year (SAMSE +1) would result in a negative stochastic growth rate, or 3‐year *r*_stoch_ of –0.0004 (SE 0.0001; Table 2). Based on average reproductive rates, SAMSE limits ranged from 2.33 to 8 individuals per year (7–24 per 3‐year), depending on the initial abundance estimate (Table 2). Deterministic growth rates (*r*_det_) were consistently greater than stochastic growth rates (Table 2).

**TABLE 2:** *Sustainable anthropogenic mortality in stochastic environments* (SAMSE) that provides the SAMSE limit (i.e., maximum number of individuals that can be removed per year without resulting in negative stochastic growth rate forecasts)

			Growth rates	
	*N_0_*	SAMSE limit	deterministic	stochastic (SE)
Mean reproduction^a^	1619	2.33	0.0004	0.0001 (0.0001)
	2953	4.33	0.0003	0.0001 (0.0001)
	5473	8	0.0003	0.0001 (0.0001)
High reproduction^a^	1619	8	0.0047	0.0008 (0.0001)
	2953	15	0.0021	0.0003 (0.0001)
	5473	28	0.0020	0.0000 (0.0001)
	***N_0_***	**SAMSE** + 1^b^	**deterministic**	**stochastic (SE)**
Mean reproduction^a^	1619	2.67	–0.0003	–0.0007 (0.0001)
	2953	4.67	–0.0001	–0.0004 (0.0001)
	5473	8.33	0.0001	–0.0003 (0.0001)
High reproduction^a^	1619	8.33	0.0019	–0.0003 (0.0001)
	2953	15.33	0.0017	–0.0003 (0.0001)
	5473	28.33	0.0018	–0.0002 (0.0001)

^a^ Results for low reproductive rates are not shown because all forecasts based on the assumption of low reproductive rates resulted in negative population growth rates.

^b^ Growth rate projections for the maximum number of dolphins that can be removed, plus 1 additional removal, resulting in negative stochastic growth rates.

## DISCUSSION

The SAMSE is an important application of PVA that uses a fully stochastic modeling approach to determine limits to human‐caused mortality of wildlife. The novelty of SAMSE lies in that it offers a PVA framework to set mortality limits by population modeling trials to detect the number of removals without causing a population decline in stochastic environments. An important feature of SAMSE is that it can readily be tailored to the population or taxon under investigation. In our case study, the flexibility of the SAMSE framework allowed us to incorporate offspring dependency on the mother's fate, a stochastic factor that had a sizeable influence on population forecasts but would be difficult to incorporate with conventional approaches.

The SAMSE limit established here for the trawl fishery‐affected dolphin population suggests that long‐term sustainability would not be achieved under any bycatch scenario considered. Aiming for sustainability would thus require a marked reduction in dolphin capture rates. Management interventions to achieve such an aim in demersal trawl fisheries include the use of bycatch reduction devices (Kennelly & Broadhurst 2021). However, our bycatch scenarios were based on dolphin capture rates reported after the introduction of these devices in all PTF nets in 2006 (Department of Fisheries, Western Australia 2010; Jaiteh et al., 2014). Our results also show that uncertainty in the abundance estimate had a relatively large effect on forecasts. Improving the SAMSE limit estimate would thus require more accurate demographic parameter estimates, especially abundance. Nevertheless, even the SAMSE limits based on the maximum abundance estimate were lower than the reported bycatch rates. Therefore, to attain viability of the population, actions to lower bycatch rates would need to be implemented well before reassessments are completed. We recommend that SAMSE limit be used to guide further management interventions to reach sustainable mortality limits.

Rather than offering a definitive number, we report SAMSE limits as a range of values, which reflects the uncertainty and variation in demographic parameters. The SAMSE limits indicate that, depending on population size, a maximum of 2.33–8 dolphins could be removed per year without causing a population decline in stochastic environments. This contrasts with the PBR estimate of 16.2 dolphins per annum, which does not incorporate stochastic factors and uses a general estimate of potential growth rate for cetaceans rather than demographic rates estimated for the focal population. There is some uncertainty in the estimation of the PBR value, which is typically based on the lower 80th percentile of the abundance estimate. Our estimation of *N*_min_, and thus PBR, was based on the lower 95% CI of that estimate, which would lead to a lower estimation of PBR. However, we applied an availability correction factor to the lower 95% CI, which corrected the estimate of *N*_min_ upward. This complicated our comparison of the calculated PBR limit with the results of our modeling approach. It is important to note that, as well as comparing PBR and SAMSE limits as we have done, it is possible to use both to exploit their different approaches.

Our modeling approach showed that human‐caused mortality at the PBR level would only be sustainable if reproductive rates were consistently high. Omission of such stochasticity leads to greater vulnerability than would be expected from a purely deterministic analysis (Punt et al., 2020a). Our analysis highlighted the impact of incorporating stochasticity, which substantially lowered the population forecasts. In particular, incorporating the dependency of calves on mothers resulted in more pessimistic forecasts. Also, incorporating variation in reproductive rates, which typically show large temporal fluctuations in dolphins (Blazquez et al., 2020; Manlik et al., 2016; Manlik 2019), had a substantial impact on model forecasts. While previous researchers incorporated various levels of stochasticity into PBR models (e.g., Brandon et al., 2017; Punt et al., 2018; Punt et al., 2020a), SAMSE offers a fully stochastic PVA framework. Moreover, SAMSE can be customized to the affected population to set human‐caused mortality limits. We utilized SAMSE with a goal of population stability or recovery in stochastic environments. However, for different target populations, users might have different conservation objectives, and these can be explicitly incorporated in SAMSE's PVA framework.

The original PBR equation is a useful, yet simple, deterministic tool to offer management advice based on minimal information on the nontarget species. It is considered a conservative estimation, but contrasts with SAMSE in that it does not account for stochasticity and uncertainty in demographic parameters. The PBR and SAMSE are not directly comparable because they represent different approaches. We defined the SAMSE limit as the maximum number of individuals that can be removed in a given period without resulting in negative stochastic growth rate forecasts. The SAMSE has an implicit goal of population recovery in stochastic environments. In contrast, PBR was designed with the goal of rebuilding populations within a given timeframe with a given probability and then maintaining populations at OSP. Moreover, we calculated PBR based on *R*_max_ = 0.04, the default value used for cetaceans (Wade 1998), but this would differ depending on the reference population selected, which may have an *R*_max_ that is considerably lower than the default—as was the case for the dolphin population. Thus, SAMSE aims to estimate a bycatch limit corresponding to stability, while approaches such as PBR aim to recover populations, which could imply even lower limits if PBR were applied to a population with lower *R*_max_. The difference between PBR and SAMSE is greater if the SAMSE model incorporates a carrying capacity that depresses forecast population growth. Therefore, the differing limits determined by SAMSE versus PBR are partly due to stochasticity, but also stem from different assumptions. With this in mind, we encourage practitioners of SAMSE, or any other method, to explicitly state the underlying assumptions.

The general SAMSE approach (Figure 1) includes 3 steps. First, one sets a stochastic baseline model based on parameters of the affected population and a taxonomically and demographically comparable reference population that is stable in the absence of human‐caused mortality. This is followed by incorporating demographic and environmental stochasticity by calculating standard deviations due to EV for mortality and reproductive rates measured across several periods. The final step consists of trialing scenarios that reduce mortalities until reaching the threshold at which 1 additional mortality would generate a negative stochastic population growth forecast. The SAMSE limit is, thus, the additional mortalities (compared with the baseline model in the absence of bycatch or other human‐caused mortality) that produce a nonnegative stochastic population growth. These steps can also be followed when using other stochastic PVA software, such as ramas (https://www.ramas.com/metapop‐6‐0). The SAMSE limit would change if abundance or other demographic parameters change, requiring new modeling. That is also true for PBR, especially if using the expanded operating models with other factors incorporated. Therefore, the SAMSE limit needs updating at regular intervals, when new abundance estimates become available. This is particularly important because SAMSE is reported as a range of absolute numbers, but may be implemented as a change in mortality rates in the modeling. If SAMSE limits are implemented without adjustments over time, this could trigger a feedback control when the population declines.

If data are lacking on the study population, the limitation and usefulness of SAMSE hinge on identifying a suitable reference population. Such a population would need to be taxonomically and demographically similar to the human‐affected population; be well studied, offering a reliable, ideally long‐term data set, including data on temporal variation in age‐specific mortality and reproductive rates; and be stable in the absence of human‐caused mortality. We recognize that reference populations that meet such stringent criteria are unavailable for many populations subject to human‐caused mortalities. In such cases, one must fall back on a generic calculation, such as the PBR or alternative approaches for low‐data environments (Punt et al., 2020b). However, we argue that, when relevant data are available, the SAMSE approach of incorporating uncertainty and stochasticity is warranted to improve the estimate of the sustainable removal rate. Given that surrogate data are usually from well‐studied reference populations, data availability is less of a problem than if one were to rely entirely on data from the affected populations. Also, in situations for which the data requirements for SAMSE are not met, a normative function of SAMSE is to encourage regulators to consider adjustments to the PBR parameters. For example, our results suggest that for small populations, PBR should err on the side of caution and that the *F_R_* default value of 0.5 should be lowered to account for other biases, such as stochasticity.

The SAMSE is broadly applicable to a range of taxa and might contribute to a “congruent roadmap” to address evaluating bycatch of sea turtles and seabirds, as called for by Moore et al. (2009, p. 446). Unlike marine mammals, bycatch of turtles and seabirds in the United States is governed by the Endangered Species Act (ESA) of 1973. However, the ESA has not adopted PBR and does not outline an explicit process to determine acceptable bycatch limits (Moore et al., 2009). We propose SAMSE as the tool of choice for this purpose if relevant data are available. Similarly, like PBR, SAMSE could be applied to a range of scenarios in which human‐caused mortalities of wildlife occur, such as lethal control (Runge et al., 2009), hunting (Parry et al., 2009), or wind turbines (Schippers et al., 2020). We therefore introduce SAMSE as a stochastic addition to the conventional, deterministic methods for a range of taxa affected by human‐caused mortality. The SAMSE modeling could be further expanded to incorporate additional data including, for example, ecological processes for multiple stocks (Kanaji et al., 2021).

## Supporting information

Supporting Information
Additional supporting information may be found in the online version of the article at the publisher's website.
