# Supplementary material for: A stochastic model for estimating sustainable limits to wildlife mortality in a changing world
Source: Conserv Biol. 2022 Apr 28;36(4):e13897. doi: 10.1111/cobi.13897 (PMC9542519; doi:10.1111/cobi.13897)
Supplement: Supplementary file 1 — Supporting Information Additional supporting information may be found in the online version of the article at the publisher's website. [file COBI-36-0-s001.pdf]

Supplementary Material for "A stochastic model for estimating sustainable limits to wildlife mortality in a changing world", *Conservation Biology*. doi:10.1111/cobi.13897

Oliver Manlik*, Robert C. Lacy, William B. Sherwin, Hugh Finn, Neil R. Loneragan, Simon J. Allen*

*Authors for correspondence: oliver.manlik@uaeu.ac.ae; simon.allen@bristol.ac.uk

1.) **Appendix S1:** Supplementary table showing three-year mortality rates for all scenarios (p. 2)

2.) **Appendix S2:** Supplementary table showing parameters from which input for Vortex models were derived (p. 3)

3.) **Appendix S3:** Calf-dependent setting; a description of how the VORTEX model was adjusted to simulate that calves are dependent on their mothers (p. 4)

4.) **Appendix S4:** Supplementary table showing population forecasts for scenarios based on high reproductive rates (p.5)

5.) **Appendix S5:** Supplementary table showing population forecasts for scenarios based on low reproductive rates (p. 6)

6.) **Appendix S6:** Supplementary figure showing population trajectories for scenarios based on three abundance estimates (N_0 = 2,953; N_0 = 5,473; N_0 = 1,619) (p. 7)

7.) **Appendix S7:** Supplementary table showing population forecasts when incorporating vs omitting different levels of stochasticity (p. 8)

8.) **Appendix S8:** Supplementary table showing population forecasts comparing dependent-calf versus independent-calf settings (p. 9)

9.) **References for Supplementary Material** (p. 10)

Manlik O, Lacy RC, Sherwin WB, Hugh F, Loneragan NR, Allen SJ. (2022). A stochastic model for estimating sustainable limits to wildlife mortality in a changing world. Conservation Biology. doi:10.1111/cobi.13897

Appendix S1

Table Appendix S1. Three-year mortality rates for all scenarios. Baseline population sizes taken from aerial survey estimates in Allen et al. (2017) and adjusted based on the availability correction in Forcada et al. (2004).

**Scenarios**		**Calves**	**Juve-1**[b]	**Juve-2**[b]	**Subadults**[b]	**Adults**
Baseline Scenarios[a]		26.52%/18.61%[c] (SD$_{EV}$ 3.36/2.36)	4.29% (SD$_{EV}$ 2.28)	1.06% (SD$_{EV}$ 1.23)	3.08% (SD$_{EV}$ 2.66)	9.72% (SD$_{EV}$ 1.40)
***N₀* = 2,953**	Bycatch 1*	19.93%	5.61%	2.38%	4.40%	11.04%
	Bycatch 2*	21.10%	6.78%	3.55%	5.57%	12.21%
	Bycatch 3*	23.69%	9.34%	6.11%	8.13%	14.81%
	Max Bycatch*	26.23%	11.92%	8.69%	10.71%	17.33%
***N₀* = 5,473**	Bycatch 1*	19.32%	4.99%	1.77%	3.79%	10.43%
	Bycatch 2*	19.95%	5.63%	2.40%	4.42%	11.06%
	Bycatch 3*	21.35%	7.02%	3.79%	5.81%	12.47%
	Max Bycatch*	22.72%	8.47%	5.06%	7.26%	13.83%
***N₀* = 1,619**	Bycatch 1*	20.95%	6.70%	3.47%	5.49%	12.13%
	Bycatch 2*	23.11%	8.83%	5.61%	7.62%	14.26%
	Bycatch 3*	27.84%	13.50%	10.28%	12.29%	19.01%
	Max Bycatch*	32.47%	18.42%	14.58%	17.21%	23.60%

[a]*Baseline mortality rates are from Manlik et al. (2016). SD$_{EV}$ (standard deviations due to environmental variance) as reported by Manlik et al. (2016) are shown in brackets. Note: the same SD$_{EV}$s for the respective age classes were used for each of the bycatch scenarios.*

[b]*Juvenile survival rates were subdivided into 'juve-1', 'juve-2' and 'subadults', following the convention for the Shark Bay bottlenose dolphin population (Manlik et al. 2016).*

[c]*26.52% is the observed 3-yr calf mortality rate for the Shark Bay population (Manlik et al., 2016), which was used for the model with the assumption that calf mortality is not dependent on the mortalities of mothers; 18.61% is the adjusted 3-yr calf mortality rate, which was used for the calf-dependent model. All other calf mortality rates shown are the adjusted 3-yr rates for the calf-dependent model.*

Bycatch scenarios: "Bycatch 1": 13/yr (39/3-yr) = capture rate for 2008, based on Western Australian Department of Fisheries Report (2010); "Bycatch 2": 24.5/yr (73.5/3-yr) = mean capture rate (2012-2017), based on skippers' logbooks (Gaughan et al. 2019);"Bycatch 3": 50/yr (150/3-yr) = mean capture rate (2002; 2006-2009), based on independent observer reports (Stephenson & Chidlow 2003; Allen et al. 2014); "Max Bycatch": 75/yr (225/-3-yr) = hypothetical maximum catch rate, based on statement by Western Australian Fisheries Department that "number of dolphins caught by the fishery should be < 75/yr" (Fletcher & Santoro 2010, p. 313).*

Appendix S2

Table Appendix S2. Parameters from which input for VORTEX models were derived. Mortality rates and other input values are listed in Table 1 of the main text.

**Initial Population Size (N_0)**	**N_0**	**Population**
Best estimate (corrected)	2,953	Pilbara Trawl Fishery*
Corrected lower limit of 95% CI	1,619	Pilbara Trawl Fishery*
Corrected upper limit of 95% CI	5,473	Pilbara Trawl Fishery*

**Initial age class distribution (all scenarios)**	**%**	**Population**
Calves	14.67	Shark Bay**
Juveniles	30.16	Shark Bay**
Adults	55.15	Shark Bay**

**Reproductive System**		**Population**
Female maturity at age category (age)	> 4 (> 12 yr)***	Shark Bay**
Male maturity at age category (age)	> 5 (> 15 yr)***	Shark Bay**
Maximum age category (age) at reproduction	10 (30-33 yr)***	Shark Bay**
Sex ratio at birth (male:female)	50:50	Shark Bay**
Mean % adult females breeding/3-yr	58.35% (SD_{EV} 9.38)	Shark Bay**
+ 1 SE % adult females breeding/3-yr	63.95% (SD_{EV} 9.38)	Shark Bay**
– 1 SE % adult females breeding/3-yr	52.75% (SD_{EV} 9.38)	Shark Bay**

**Data source: Allen et al. 2017 with correction factor from Forcada et al. 2004.*

***Data source: Manlik et al. 2016; SD_{EV} (standard deviations due to environmental variance) for percentage of adult females breeding as reported by Manlik et al. (2016) is shown in brackets.*

****Females and males mature on average from age 12 and 15 years, respectively (Mann et al. 2000). This corresponds with the 3-year VORTEX input age category of 4 (females) and 5 (males). One Vortex "year" was stipulated to be 3 calendar years (i.e.1095 days).*

Appendix S3: Calf-dependent setting

Bottlenose calves are dependent on their mothers until weaned (Wells et a. 1987; Mann et al. 2000), so if a mother is killed in a net, its calf is unlikely to survive—either because it is also caught in the net due to its close association with the mother or because it will not survive to weaning thereafter. We therefore adjusted the model to make calves dependent on their mothers. We set up an "individual state variable" in VORTEX, which is used to track how many dependent offspring each female has at any giving time period. For the "initialization function", we entered "0" because, at the outset of the simulation, offspring are not yet assigned to known females, and 0 for the "birth function". The transition function was set to = IS1, stipulating that VORTEX controls the value, instead of the function changing it. Also, see the VORTEX manual for detials of implementing this option (Lacy et al. 2014). This setting also allowed us to stipulate that females do not reproduce again until the prior offspring is independent, in accord with the reproductive cycle of bottlenose dolphins (Mann et al. 2000).

Making offspring survival dependent on dams also required us to adjust calf mortality. The average observed 3-yr calf mortality rate in the absence of bycatch was 26.52% (Manlik et al. 2016). This included mortalities due to the death of the mother. Therefore, we first adjusted the calf mortality entered in the model to exclude the component of calf mortality due to the mother's death from the total mortality rate entered. We thus calculated the 3-yr calf mortality rate not due to the death of the mother (*Mc*'): *Mc*' = 1 – (1 – *Mc*)/(1 – *Ma*), where *Mc* = total calf mortality and *Ma* = adult female mortality. Subsequently, we applied this adjusted 3-yr calf mortality rate of 18.61% and compared the forecasts based on the models with and without the assumption of the calves' dependency on their mothers. As expected, the forecast of the calf-dependent standard mirrored that of the standard calf-independent model (i.e. in the absence of bycatch) (see Results, main text).

Appendix S4

Table Appendix S4. High reproduction: Population forecasts for N_0 = 2,953 (best estimate), N_0 = 5,473 and N_0 = 1,619. Three-year stochastic growth rates (r_{stoch}), percentage change in *N* and mean time to extinction for all scenarios based on high reproductive rates. Standard errors for r_{stoch}. and *N* are shown in parentheses. The 63.3-year forecasts represent forecasts for three *Tursiops* spp. generations (Taylor et al. 2007).

**N_0 = 2,953**		***No bycatch***	**Bycatch 1***	**Bycatch 2***	**Bycatch 3***	**Max Bycatch***
Stochastic growth rate $r_{stoch.}$		**0.0196** (0.0001)	**0.00206** (0.0001)	**-0.0116** (0.0001)	**-0.0487** (0.0002)	**-0.0866** (0.0004)
Time to extinction (yrs)		NA	NA	NA	282.9	234.0
**63.3-yr forecast**	$N_{63.3}$	4,594.5 (23.9)	3,293.9 (17.6)	2,459.8 (14.1)	1,213.9 (6.9)	595.3 (3.4)
	% Change	55.6%	11.5%	-16.7%	**-58.9%**	**-79.8%**
**100-yr forecast**	N_{100}	5,263 (30.3)	3387.7 (23.1)	2,144.6 (16.1)	686.7 (5.2)	223.7 (1.9)
	% Change	78.3%	14.7%	-27.4%	-76.7%	-92.4%
**N_0 = 5,473**		***No bycatch***	**Bycatch 1***	**Bycatch 2***	**Bycatch 3***	**Max Bycatch***
Stochastic growth rate $r_{stoch.}$		**0.0196** (0.0001)	**0.0104** (0.0001)	**0.0026** (0.0001)	**-0.0155** (0.0001)	**-0.0335** (0.0001)
Time to extinction (yrs)		NA	NA	NA	NA	NA
**63.3-yr forecast**	$N_{63.3}$	8,013.3 (32.5)	5,508.1 (11.17)	5057.6 (28.7)	4,228.8 (23.0)	2,927.0 (16.2)
	% Change	46.4%	0.6%	-7.6%	-22.7%	-46.5%
**100-yr forecast**	N_{100}	8,436.9 (33.9)	5,285.3 (32.5)	4768.5 (30.0)	3,518.0 (25.6)	1,958 (14.3)
	% Change	54.2%	-3.43%	-12.9%	-35.7%	-64.2%
**N_0 = 1,619**		***No bycatch***	**Bycatch 1***	**Bycatch 2***	**Bycatch 3***	**Max Bycatch***
Stochastic growth rate $r_{stoch.}$		**0.0195** (0.0001)	**-0.0094** (0.0003)	**-0.0412** **(**0.0002)	**-0.1099** (0.0005)	**-0.1717** (0.0007)
Time to extinction (yrs)		NA	NA	282.5	172.4	111.4
**63.3-yr forecast**	$N_{63.3}$	2,563 (13.2)	1,432.3 (25.7)	764.9 (4.6)	203.0 (1.4)	51.2 (0.5)
	% Change	58.3%	-11.5%	**-52.8%**	**-87.5%**	**-96.8%**
**100-yr forecast**	N_{100}	3,245 (21.5)	1,271.9 (29.5)	477.5 (4.0)	56.8 (0.7)	7.6 (0.2)
	% Change	100.4%	-21.4%	-70.5%	-96.5%	-99.5%
	*PE** (%)	0.0%	0.0%	0.0%	0.0%	27.5%

Bycatch scenarios: "Bycatch 1": 13/yr (39/3-yr) = capture rate for 2008, based on Western Australian Department of Fisheries Report (2010); "Bycatch 2": 24.5/yr (73.5/3-yr) = mean capture rate (2012-2017), based on skippers' logbooks (Gaughan et al. 2019);"Bycatch 3": 50/yr (150/3-yr) = mean capture rate (2002; 2006-2009), based on independent observer reports (Stephenson & Chidlow 2003; Allen et al. 2014); "Max Bycatch": 75/yr (225/-3-yr) = hypothetical maximum catch rate, based on statement by Western Australian Fisheries Department that "number of dolphins caught by the fishery should be < 75/yr" (Fletcher & Santoro 2010, p. 313). Probability of extinction (PE) shows the percentage of iterations for which the population was forecast to go extinct. PE was only tabulated for rows that included PE values that were greater than 0%.*

Appendix S5

Table Appendix S5. Low reproduction: Population forecasts for N_0 = 2,953 (best estimate), N_0 = 5,473 and N_0 = 1,619. Three-year stochastic growth rates (r_{stoch}), percentage change in N and mean time to extinction for all scenarios based on low reproductive rates. Standard errors for $r_{stoch.}$ and N are shown in parentheses. The 63.3-year forecasts represent forecasts for three *Tursiops* spp. generations (Taylor et al. 2007).

**N_0 = 2,953**		***No bycatch***	**Bycatch 1***	**Bycatch 2***	**Bycatch 3***	**Max Bycatch***
Stochastic growth rate $r_{stoch.}$		**-0.0099** (0.0001)	**-0.0267** (0.0001)	**-0.0423** (0.0002)	**-0.0811** (0.0003)	**-0.1137** (0.0004)
Time to extinction (yrs)		NA	222.0	283.4	245.7	182.7
**63.3-yr forecast**	$N_{63.3}$	2,572.9 (14.2)	1,812.6 (10.8)	1,355.1 (8.1)	664.1 (4.1)	329.8 (2.1)
	% Change	-12.9%	-38.6%	**-54.1%**	**-77.5%**	**-88.8%**
**100-yr forecast**	N_{100}	2,289.0 (16.8)	1,324.7 (10.5)	829.5 (6.8)	268.2 (2.3)	87.1 (0.9)
	% Change	-22.5%	-55.1%	-71.9%	-91.0%	-97.0%
**N_0 = 5,473**		***No bycatch***	**Bycatch 1***	**Bycatch 2***	**Bycatch 3***	**Max Bycatch***
Stochastic growth rate $r_{stoch.}$		**-0.0097** (0.0001)	**-0.0187** (0.0001)	**-0.0270** (0.0001)	**-0.0459** (0.0001)	**-0.0664** (0.0002)
Time to extinction (yrs)		NA	NA	NA	283.2	287.8
**63.3-yr forecast**	$N_{63.3}$	4,731.3 (25.5)	3,714.0 (21.8)	3,359.1 (18.8)	2.296.9 (13.2)	1,589.0 (9.1)
	% Change	-13.6%	-32.1%	-38.6%	**-58.0%**	**-71.0%**
**100-yr forecast**	N_{100}	4,219.2 (30.6)	2,936.2 (22.2)	2,430.9 (18.3)	1,325.4 (10.0)	745.0 (6.2)
	% Change	-22.9%	-46.4%	-55.6%	-75.8%	-86.4%
**N_0 = 1,619**		***No bycatch***	**Bycatch 1***	**Bycatch 2***	**Bycatch 3***	**Max Bycatch***
Stochastic growth rate $r_{stoch.}$		**-0.0100** (0.0001)	**-0.0401** (0.0005)	**-0.0739** (0.0003)	**-0.1361** (0.0005)	**-0.1964** (0.0007)
Time to extinction (yrs)		NA	279.6	242.1	139.5	97.6
**63.3-yr forecast**	$N_{63.3}$	1,399 (27.6)	783.0 (14.9)	426.8 (2.8)	112.5 (0.9)	28.9 (0.3)
	% Change	-13.5%	**-51.5%**	**-73.6%**	**-93.0%**	**-98.2%**
**100-yr forecast**	N_{100}	1,257.8 (10.3)	491.4 (11.9)	187.0 (1.7)	22.2 (0.3)	4.9 (0.2)
	% Change	-22.3%	-69.7%	-88.4%	-98.6%	-99.7%
	*PE** (%)	0.0%	0.0%	0.0%	0.5%	66.1%

Bycatch scenarios: "Bycatch 1": 13/yr (39/3-yr) = capture rate for 2008, based on Western Australian Department of Fisheries Report (2010); "Bycatch 2": 24.5/yr (73.5/3-yr) = mean capture rate (2012-2017), based on skippers' logbooks (Gaughan et al. 2019);"Bycatch 3": 50/yr (150/3-yr) = mean capture rate (2002; 2006-2009), based on independent observer reports (Stephenson & Chidlow 2003; Allen et al. 2014); "Max Bycatch": 75/yr (225/-3-yr) = hypothetical maximum catch rate, based on statement by Western Australian Fisheries Department that "number of dolphins caught by the fishery should be < 75/yr" (Fletcher & Santoro 2010, p. 313). Probability of extinction (PE) shows the percentage of iterations for which the population was forecast to go extinct. PE was only tabulated for rows that included PE values that were greater than 0%.*

Appendix S6

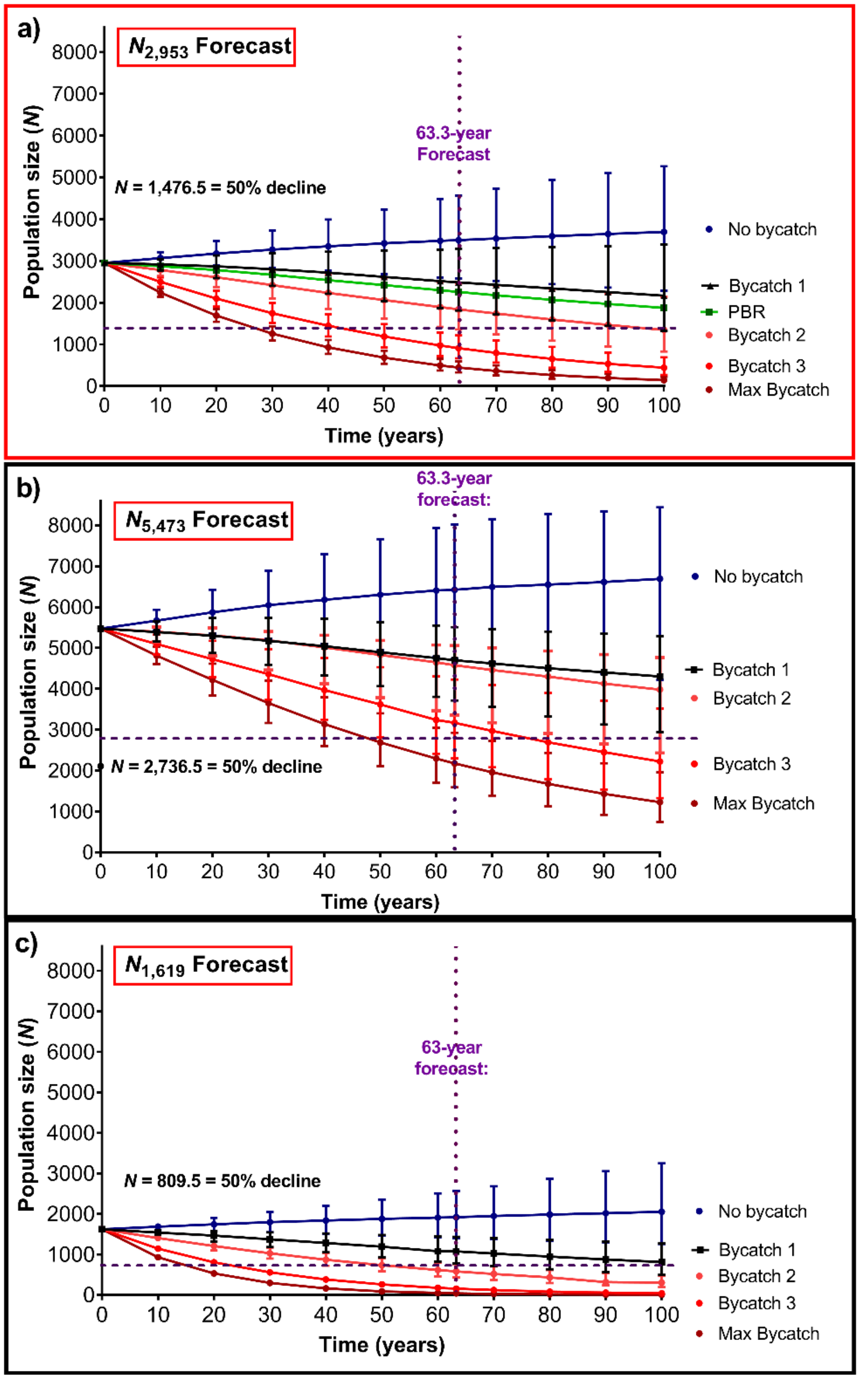

*"**Bycatch 1**": 13/yr (39/3-yr) = capture rate for 2008, based on Western Australian Fisheries Department Report (2010);*

*"**Bycatch 2**": 24.5/yr (73.5/3-yr) = mean capture rate (2012-2017), based on skippers' logbooks (Gaughan et al. 2019);*

*"**Bycatch 3**": 50/yr (150/3-yr) = mean capture rate (2002; 2006-2009), based on independent observer reports (Stephenson & Chidlow 2003; Allen et al. 2014);*

*"**Max Bycatch**": 75/yr (225/-3-yr) = hypothetical maximum catch rate, based on statement by Western Australian Fisheries Department that "number of dolphins caught by the fishery should be < 75/yr" (Fletcher & Santoro 2010, p. 313).*

Supplementary Figure Appendix S6. Dolphin population trajectories based on four baseline scenarios without bycatch-related mortalities (blue) and bycatch scenarios based on 39, 73.5, 150, 225 and 48.57 (PBR; green) dolphin captures per 3-yr period generated from models with three initial population sizes: a) N_0 = 2,953; b) N_0 = 5,473; c) N_0 = 1,619. Whiskers depict the results for high (+ 1 SE) and low (-1 SE) reproductive rates (Manlik et al. 2016). Note that panel a) shows the same trajectory as shown in Figure 2 of the main text, i.e. for the model based on the best abundance estimate (N_0 = 2,953).

Appendix S7

Table Appendix S7. 3-year population growth (*r*) forecasts, based on bycatch scenarios with varying levels of stochasticity: Stochastic model forecasts are based on the fully stochastic model, as reported in Table 1; 0 SD_{EV} lists the forecasts, based on scenarios with 0 standard deviations due to environmental variance (SD_{EV}) for each the percentage of adult females breeding (‘Repro’), all age-specific mortalities (‘Mort’) and both (‘Repro & Mort’); The deterministic calculation lists 3-yr growth rates in the absence of stochastic factors (VORTEX, v10; Lacy et al. 2014). The percentage change of mean 3-year growth rates relative to those based on deterministic calculations are listed in the ‘% Change vs Det’ rows.

N_0 = 2,953

Scenarios*	Stochastic Model	**0 SD_{EV}**			Deterministic Calculation
		Repro	Mort	Repro & Mort	
Bycatch 1	-0.0115	-0.0101	-0.0103	-0.0101	-0.0096
Bycatch 2	-0.0262	-0.0240	-0.0236	-0.0230	-0.0229
Bycatch 3	-0.0650	-0.0567	-0.0562	-0.0554	-0.0528
Max Bycatch	-0.1002	-0.0859	-0.0866	-0.0863	-0.0802
MEAN	-0.0507	-0.0442	-0.0442	-0.0437	-0.0414
% Change vs Det.	-22.6%	-6.8%	-6.8%	-5.6%	0.0%

N_0 = 5,473

Scenarios*	Stochastic Model	**0 SD_{EV}**			Deterministic Calculation
		Repro	Mort	Repro & Mort	
Bycatch 1	-0.0034	-0.0030	-0.0031	-0.0029	-0.0027
Bycatch 2	-0.0116	-0.0101	-0.0101	-0.0100	-0.0098
Bycatch 3	-0.0294	-0.0264	-0.0260	-0.0257	-0.0257
Max Bycatch	-0.0486	-0.0411	-0.0410	-0.0411	-0.0403
MEAN	-0.0233	-0.0202	-0.0201	-0.0199	-0.0196
% Change vs Det.	-18.4%	-2.6%	-2.1%	-1.5%	0.0%

N_0 = 1,619

Scenarios*	Stochastic Model	**0 SD_{EV}**			Deterministic Calculation
		Repro	Mort	Repro & Mort	
Bycatch 1	-0.0241	-0.0213	-0.0213	-0.0209	-0.0203
Bycatch 2	-0.0603	-0.0533	-0.0493	-0.0490	-0.0467
Bycatch 3	-0.1211	-0.1108	-0.1096	-0.1076	-0.1033
Max Bycatch	-0.1834	-0.1692	-0.1654	-0.1665	-0.1625
MEAN	-0.0972	-0.0887	-0.0864	-0.0860	-0.0832
% Change vs Det.	-16.9%	-6.6%	-3.8%	-3.4%	0.0%

**Bycatch scenarios: “Bycatch 1”: 13/yr (39/3-yr) = capture rate for 2008, based on Western Australian Department of Fisheries Report (2010); “Bycatch 2”: 24.5/yr (73.5/3-yr) = mean capture rate (2012-2017), based on skippers’ logbooks (Gaughan et al. 2019);“Bycatch 3”: 50/yr (150/3-yr) = mean capture rate (2002; 2006-2009), based on independent observer reports (Stephenson & Chidlow 2003; Allen et al. 2014); “Max Bycatch”: 75/yr (225/-3-yr) = hypothetical maximum catch rate, based on statement by Western Australian Fisheries Department that “number of dolphins caught by the fishery should be < 75/yr” (Fletcher & Santoro 2010, p. 313).*

Appendix S8

Table Appendix S8. 3-year stochastic population growth (r_{stoch}) forecasts for calves whose fate is not dependent on their mothers' mortality ('Independent-Calf') versus calves whose fate is dependent on their mother's mortality ('Dependent-Calf') for N_0 = 2,953. The percentage change of mean 3-year growth rate for the dependent-calf model (-0.0047) relative to the independent-calf model (-0.0507) is shown in the '% Change vs Ind.' row.

Scenarios*	Independent-Calf	Dependent-Calf
Bycatch 1	-0.0104	-0.0115
Bycatch 2	-0.0233	-0.0262
Bycatch 3	-0.0573	-0.0650
Max Bycatch	-0.0878	-0.1002
MEAN	-0.0447	-0.0507
% Change vs Ind.	-13.4%	

**Bycatch scenarios: "Bycatch 1": 13/yr (39/3-yr) = capture rate for 2008, based on Western Australian Department of Fisheries Report (2010); "Bycatch 2": 24.5/yr (73.5/3-yr) = mean capture rate (2012-2017), based on skippers' logbooks (Gaughan et al. 2019);"Bycatch 3": 50/yr (150/3-yr) = mean capture rate (2002; 2006-2009), based on independent observer reports (Stephenson & Chidlow 2003; Allen et al. 2014); "Max Bycatch": 75/yr (225/-3-yr) = hypothetical maximum catch rate, based on statement by Western Australian Fisheries Department that "number of dolphins caught by the fishery should be < 75/yr" (Fletcher & Santoro 2010, p. 313).*

Literature Cited

Allen SJ, Tyne JA, Kobryn HT, Bejder L, Pollock KH, Loneragan NR. 2014. Patterns of dolphin bycatch in a north-western Australian trawl-fishery. PLOS ONE:9(e93178). doi:10.1371/journal.pone.0093178

Department of Fisheries, Western Australia. 2010. The bycatch action plan for the Pilbara fish trawl interim managed fisheries. Fisheries Management Paper No. 244. ISSN 0819-4327.

Fletcher WJ, Santoro K. 2010. State of the Fisheries and Aquatic Resources Report 2009/10. Department of Fisheries, Western Australia.

Forcada J, Gazo M, Aguilar A, Gonzalvo J, Fernandez-Contreras M. 2004. Bottlenose dolphin abundance in the NW Mediterranean: addressing heterogeneity in distribution. Marine Ecology Progress Series 275:275–287.

Gaughan DJ, Molony B, Santoro K. 2019. *Status Reports of the Fisheries and Aquatic Resources of Western Australia 2017/18: The State of the Fisheries.* Department of Primary Industries and Regional Development, Western Australia.

Lacy RC, Miller PS, Traylor-Holzer K. 2014. Vortex 10 User's Manual. IUCN SSC Conservation Breeding Specialist Group, and Chicago Zoological Society, Apple Valley, Minnesota, USA.

Manlik O, McDonald JA, Mann J, Raudino HC, Bejder L, Krützen M, Connor RC, Heithaus MR, Lacy RC, Sherwin WB. 2016. The relative importance of reproduction and survival for the conservation of two dolphin populations. Ecology and Evolution 6(11):3496-3512. doi:10.1002/ece3.2130.

Mann J, Connor RC, Barré LM, Heithaus MR. 2000. Female reproductive success in bottlenose dolphins (*Tursiops* sp.): life history, habitat, provisioning, and group-size effects. Behavioral Ecology 11(2):210-219.

Stephenson PC, Chidlow J. 2003. Bycatch in the Pilbara trawl fishery. Final report to the Natural Heritage Trust. Perth: Natural Heritage Trust. 74 p.

Taylor BL, Chivers SJ, Larse J, Perrin WF. 2007. Generation length and percent mature estimates for IUCN assessments of cetaceans. Administrative Report LJ-07-01. National Marine Fisheries Services, Southwest Fisheries Science Center, U.S.A.

Wells RS, Scott MD, Irvine AB. 1987. The social structure of free-ranging bottlenose dolphins. Pages 247-305 in Genoways HH, editor. Current Mammalogy. Springer, Boston, MA.
